# Effect of obesity on intraoperative bleeding volume in open gastrectomy with D2 lymph-node dissection for gastric cancer

**DOI:** 10.1186/1754-9493-2-7

**Published:** 2008-04-24

**Authors:** Hirochika Makino, Chikara Kunisaki, Hirotoshi Akiyama, Hidetaka A Ono, Takashi Kosaka, Ryo Takagawa, Yasuhiko Nagano, Syoichi Fujii, Hiroshi Shimada

**Affiliations:** 1 Department of Surgery, Gastroenterological Center, Yokohama City University, 4-57, Urafune-cho, Minami-ku, Yokohama, 232-0024, Japan; 2 Department of Gastroenterological Surgery, Yokohama City University School of Medicine, 3-9 Fukuura, Kanazawa-ku, Yokohama, 236-0004, Japan

## Abstract

**Background:**

To investigate the effect of obesity on open gastrectomy with D2 lymph-node dissection.

**Methods:**

Between January 2005 and March 2007, 100 patients with preoperatively diagnosed gastric cancer who underwent open gastrectomy with D2 lymph-node dissection were enrolled in this study. Of these, 61 patients underwent open distal gastrectomy (ODG) and 39 patients underwent open total gastrectomy (OTG). Patients were classified as having a high body-mass index (BMI; ≥ 25.0 kg/m^2^; *n *= 21) or a normal BMI (<25.0 kg/m^2^; *n *= 79). The visceral fat area (VFA) and subcutaneous fat area (SFA) were assessed as identifiers of obesity using FatScan software. Patients were classified as having a high VFA (≥ 100 cm^2^; *n *= 34) or a normal VFA (<100 cm^2^; *n *= 66). The relationship between obesity and short-term patient outcomes after open gastrectomy was evaluated. Patients were classified as having high intraoperative blood loss (IBL; ≥ 300 ml; *n *= 42) or low IBL (<300 ml; *n *= 58). Univariate and multivariate analyses were used to identify predictive factors for high IBL.

**Results:**

Significantly increased IBL was seen in the following: patients with high BMI versus normal BMI; patients with gastric cancer in the upper third of the stomach versus gastric cancer in the middle or lower third of the stomach; patients who underwent OTG versus ODG; patients who underwent splenectomy versus no splenectomy; and patients with high VFA versus low VFA. BMI and VFA were significantly greater in the high IBL group than in the low IBL group. There was no significant difference in morbidity between the high IBL group and the low IBL group. Multivariate analysis revealed that patient age, OTG and high BMI or high VFA independently predicted high IBL.

**Conclusion:**

It is necessary to perform operative manipulations with particular care in patients with high BMI or high VFA in order to reduce the IBL during D2 gastrectomy.

## Background

Obesity is associated with substantial technical difficulties and increased patient morbidity after open gastrectomy [1,2]. The body-mass index (BMI) has been widely used as an indicator of the extent of obesity in patients [1-3]. However, the BMI does not always accurately reflect the volume of visceral fat, because the distribution of fatty tissue differs greatly between individuals [4].

Recently, several techniques have been developed to assess the volume of visceral fat. In terms of reproducibility and accuracy, computed tomography (CT) is considered to be the optimal technique for assessing visceral fat [5] compared with alternatives such as ultrasonography [6], magnetic-resonance imaging [7] and other anthropometric measurements [8,9].

Several studies have shown that the visceral fat area (VFA) determined from a single scan at the level of the umbilicus is closely correlated with the total volume of visceral fat [10,11]. The VFA might therefore accurately reflect the extent of obesity in patients.

Few previous reports have evaluated the association between the VFA and technical difficulties during gastrectomy with D2 lymph-node dissection for gastric cancer. To address this issue, we examined the influence of the VFA on gastrectomy with D2 lymph-node dissection.

## Methods

Between January 2005 and March 2007, 100 consecutive patients with a preoperative diagnosis of gastric cancer who underwent open gastrectomy with D2 lymph-node dissection at the Department of Surgery, Gastroenterological Centre, Yokohama City University, Japan, were enrolled in this study. In total, 61 patients underwent open distal gastrectomy (ODG) and 39 patients underwent open total gastrectomy (OTG). The participants comprised 72 men and 28 women, and were aged between 36 and 85 years (mean ± standard deviation (SD) = 66.0 ± 9.8 years). All of the patients were confirmed as having gastric adenocarcinoma following endoscopic biopsies. All of the patients also underwent a barium-swallow study and CT scans. Patient data were retrieved from operative and pathological reports.

The staging and definition of lymph nodes were principally based on the Japanese Classification of Gastric Carcinoma (JGC) [12]. Experienced pathologists were employed to ensure a high quality of pathological diagnosis. Surgery was performed after all of the possible alternative procedures and treatments had been explained to each patient, and informed consent had been obtained.

All of the patients underwent D2 lymph-node dissection of gastric cancer, as defined by the JGC. The standard reconstruction methods were Billroth I gastroduodenostomy after ODG and Roux-en-Y oesophagojejunostomy after OTG. In total, 50 patients underwent gastrectomy using the LigaSure^TM^ Atlas (Valleylab, Boulder, CO) to seal all of the lymphatic ducts and vessels without suture ligation (the LigaSure group). The remaining 50 patients underwent gastrectomy using the conventional surgical approach, in which most of the lymphatic ducts and vessels were ligated with sutures (the non-LigaSure group).

To assess the influence of BMI on open gastrectomy, the patients were classified into two groups: a high BMI group (BMI ≥ 25 kg/m^2^; *n *= 21) and a normal BMI group (BMI <25 kg/m^2^; *n *= 79). The patient characteristics, intraoperative parameters and postoperative parameters were compared between the two groups.

The mean intraoperative blood loss (IBL) was 345.2 ± 273.7 ml. To assess the influence of obesity on open gastrectomy, the patients were divided into two groups according to IBL: a high IBL group (IBL ≥ 300 ml; *n *= 42) and a low IBL group (IBL <300 ml; *n *= 58). The patient characteristics, intraoperative parameters and postoperative parameters were compared between the two groups.

The patients were also classified into two groups based on the VFA in accordance with the Japan Society for the Study of Obesity [13]: a high VFA group (≥ 100 cm^2^; *n *= 34) and a normal VFA group (<100 cm^2^; *n *= 66).

Logistic regression analysis was performed to evaluate the risk factors for high IBL.

### Fat volume

**Figure 1:**
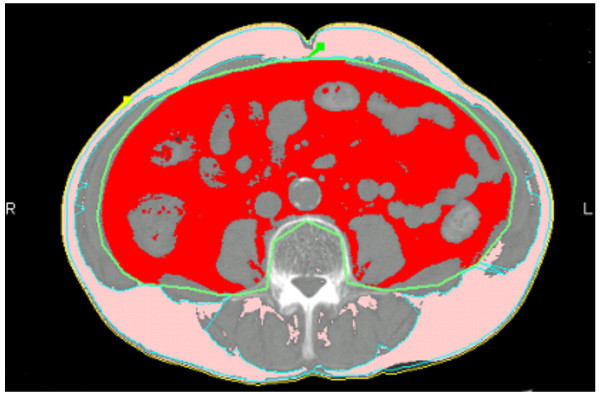
**Distribution of abdominal fat as measured by FatScan software on a CT scan at the umbilicus level.** The visceral fat area was regarded as red, and the subcutaneous fat area was regarded as pink.

The subcutaneous fat area (SFA) and the VFA were preoperatively measured (both in cm^2^) using a cross-sectional CT scan at the level of the umbilicus by FatScan software version 3 (N2 systems Inc., Osaka, Japan; Figure 1).

### Statistical analysis

The SPSS program version 10.0 for Windows (SPSS Inc, Chicago, IL) was used for all statistical analyses. A Chi-square test was applied to evaluate the differences in the proportions of each variable. The Student's *t*-test was used to evaluate the continuous variables. All data are expressed as the mean ± SD. Logistic-regression analysis was performed to evaluate the predictive factors for high IBL using the following 10 variables: age (years); gender (male vs. female); tumour location (upper third vs. middle or lower third); macroscopic type (superficial vs. well-defined vs. ill-defined); tumour size (mm); histological type (differentiated vs. undifferentiated); lymph-node metastasis (N0 vs. N1 vs. N2 vs. N3); preoperative complications (absence vs. presence); LigaSure^TM^ Atlas (not used vs. used); and either BMI (low vs. high) or VFA (low vs. high). A *p *value <0.05 was regarded as statistically significant.

## Results

### Patient characteristics and BMI

There were significant differences in the SFA and the VFA between the high BMI group and the normal BMI group. There were no differences in any other clinicopathological factors between these two groups (Table 1).

**Table 1:** Patient characteristics and BMI undergoing open gastrectomy with D2 lymph-node dissection for gastric cancer

	High BMI (*n *= 21) (%)	Normal BMI (*n *= 79) (%)	*p *value
Age (years)	67.5 ± 6.0	65.6 ± 10.6	0.4361
Gender			0.1153
Male	18 (85.7)	54 (68.4)	
Female	3 (14.3)	25 (31.6)	
SFA (cm^2^)	155.2 ± 35.8	105.3 ± 53.4	0.0001
VFA (cm^2^)	155.0 ± 41.1	69.5 ± 41.7	<0.0001
Tumor location			0.5773
U	2 (9.5)	10 (12.7)	
M	12 (57.1)	35 (44.3)	
L	7 (33.3)	34 (43.0)	
Macroscopic type			0.6873
Superficial	6 (28.6)	25 (31.6)	
Well-defined	9 (42.9)	26 (32.9)	
Ill-defined	6 (28.6)	28 (35.4)	
Tumor size (mm)	50.1 ± 35.8	56.4 ± 27.9	0.3898
Histological type			0.9490
Differentiated	11 (52.4)	42 (53.2)	
Undifferentiated	10 (47.6)	37 (46.8)	
Depth of invasion			0.5844
T1	5 (23.8)	28 (41.2)	
T2	9 (42.9)	16 (23.5)	
T3	7 (33.3)	24 (35.3)	
Lymph-node metastasis			0.4651
N0	7 (33.3)	28 (40.0)	
N1	9 (42.9)	27 (38.6)	
N2	5 (23.8)	12 (17.1)	
N3	0 (0)	3 (4.3)	
Stage			0.5214
IA	4 (19.0)	18 (22.8)	
IB	4 (19.0)	18 (22.8)	
II	3 (14.3)	19 (24.1)	
IIIA	6 (28.6)	11 (13.9)	
IIIB	3 (14.3)	6 (7.6)	
IV	1 (4.8)	7 (8.9)	
Preoperative complications			0.9055
Absence	12 (57.1)	44 (55.7)	
Presence	9 (42.8)	35 (44.3)	
LigaSure Atlas			0.4614
Used	12 (57.1)	38 (48.1)	
Not used	9 (42.9)	41 (51.9)	
Operative procedure			0.5091
Distal gastrectomy	8 (53.3)	53 (62.4)	
Total gastrectomy	7 (46.7)	32 (37.6)	
Splenectomy			0.9817
Absence	16 (76.2)	60 (75.9)	
Presence	5 (23.8)	19 (24.1)	

BMI = body-mass index; SFA = subcutaneous fat area; VFA = visceral fat area;

P < .05 was considered statistically significant

### Surgical outcomes and BMI

The IBL was significantly greater in the high BMI group than in the normal BMI group. By contrast, the operating time, the number of dissected lymph nodes and the morbidity did not differ between the two groups. No hospital death occurred among the patients (Table 2).

**Table 2:** Surgical outcomes and BMI undergoing open gastrectomy with D2 lymph-node dissection for gastric cancer

	High BMI (*n *= 21) (%)	Normal BMI (*n *= 79) (%)	*p *value
Operating time (min)	262.7 ± 83.6	245.4 ± 77.9	0.3752
Estimated blood loss (ml)	454.9 ± 303.0	316.0 ± 259.7	0.0381
Number of dissected lymph nodes	33.7 ± 11.8	37.9 ± 18.8	0.3316
Morbidity			0.1673
Absence	16 (76.2)	70 (88.6)	
Presence	5 (23.8)	9 (11.4)	
Hospital death	0	0	1.0000

BMI = body-mass index; P < .05 was considered statistically significant

### Patient characteristics and IBL

The patient age was significantly greater (*p *= 0.0318) in the high IBL group than in the low IBL group. The BMI and the VFA were significantly greater (*p *= 0.0308 and *p *= 0.0106, respectively) in the high IBL group than in the low IBL group. The tumour location, the operative procedure and the incidence of splenectomy differed significantly (*p *= 0.002, *p *= 0.0015 and *p *<0.0001, respectively) between the two groups. There were no differences in any other clinicopathological factors (Table 3).

**Table 3:** Patient characteristics and IBL undergoing open gastrectomy with D2 lymph-node dissection for gastric cancer

	High IBL (*n *= 42) (%)	Low IBL (*n *= 58) (%)	*p *value
Age (years)	68.5 ± 8.1	64.2 ± 10.6	0.0318
Gender			0.4271
Male	32 (76.2)	40 (70.0)	
Female	10 (23.8)	18 (30.0)	
BMI (kg/m^2^)			0.0100
<25	28 (66.7)	51 (87.9)	
≥25	14 (33.3)	7 (12.1)	
VFA (cm^2^)			0.0041
<100	21 (50.0)	45 (77.6)	
≥100	21 (50.0)	13 (22.4)	
SFA (cm2)	123.1 ± 53.6	110.5 ± 54.2	0.2530
Tumor location			0.0020
U	10 (23.8)	2 (3.4)	
M/L	32 (76.2)	56 (96.6)	
Macroscopic type			0.8455
Superficial	14 (33.3)	17 (29.3)	
Well-defined	15 (35.7)	20 (34.5)	
Ill-defined	13 (31.0)	21 (36.2)	
Tumor size (mm)	60.8 ± 33.2	51.0 ± 26.3	0.1016
Histological type			0.3589
Differentiated	20 (47.6)	33 (56.9)	
Undifferentiated	22 (52.4)	25 (43.1)	
Depth of invasion			0.1134
T1	12 (28.6)	21 (36.2)	
T2	20 (47.6)	16 (27.6)	
T3	10 (23.8)	21 (36.2)	
Lymph-node metastasis			0.1364
N0	15 (35.7)	29 (50.0)	
N1	16 (38.1)	20 (34.4)	
N2	8 (19.0)	9 (15.5)	
N3	3 (7.1)	0 (0)	
Stage			0.3351
IA	8 (19.0)	14 (24.1)	
IB	7 (16.7)	15 (25.9)	
II	11 (26.2)	11 (19.0)	
IIIA	6 (14.3)	11 (19.0)	
IIIB	4 (9.5)	5 (8.6)	
IV	6 (9.5)	2 (3.4)	
Preoperative complications			0.8447
Absence	24 (57.1)	32 (55.2)	
Presence	18 (42.9)	18 (31.0)	
LigaSure Atlas			0.1050
Used	17 (40.5)	33 (56.9)	
Not used	25 (59.5)	25 (43.1)	
Operative procedure			0.0015
Distal gastrectomy	24 (57.1)	43 (74.1)	
Total gastrectomy	18 (42.9)	15 (25.9)	
Splenectomy			<0.0001
Absence	24 (57.1)	52 (89.7)	
Presence	18 (42.9)	6 (10.3)	

IBL = intraoperative blood loss; BMI = body-mass index; SFA = subcutaneous fat area; VFA = visceral fat area;

P < .05 was considered statistically significant

### Surgical outcomes and IBL

The operating time was significantly longer in the high IBL group than in the low IBL group. By contrast, the number of dissected lymph nodes and morbidity did not differ between the two groups. Hospital death was not observed among the patients (Table 4).

**Table 4:** Surgical outcomes and IBL undergoing open gastrectomy with D2 lymph-node dissection for gastric cancer

	High IBL (*n *= 42) (%)	Low IBL (*n *= 58) (%)	*p *value
Operating time (min)	291.9 ± 87.4	218.1 ± 55.0	<0.0001
Dissected lymph node	37.1 ± 19.4	36.9 ± 16.4	0.9454
Morbidity			0.5131
Absence	35 (83.3)	51 (87.9)	
Presence	7 (16.7)	7 (12.1)	
Hospital death	0	0	1.0000

IBL = intraoperative blood loss; P < .05 was considered statistically significant

### Predictive factors for high IBL

Univariate analysis revealed that patient age, high BMI, high VFA, gastric cancer in the upper third of the stomach, operative procedure and use of splenectomy were associated with high IBL (Table 3). The multivariate analysis including BMI (low vs. high) along with the other nine variables described above showed that patient age, operative procedure and high BMI independently affected high IBL (*p *= 0.033 and odds ratio (OR) = 1.057, *p *= 0.001 and OR = 4.735, and *p *= 0.011 and OR = 4.356, respectively; Table 5). The multivariate analysis including VFA (low vs. high) along with the other nine variables described above showed that patient age, operative procedure and high VFA independently affected high IBL (*p *= 0.035 and OR = 1.055, *p *= 0.003 and OR = 4.071, and *p *= 0.015 and OR = 3.170, respectively; Table 6).

**Table 5:** Logistic regression analysis was performed to evaluate predictive factors for high IBL

Variable	Coefficient	Odds ratio (95% CI)	*p *value
Age	0.055	1.057 (1.005–1.112)	0.033
Operative procedure			
TG/DG	1.555	4.735 (1.832–12.238)	0.001
BMI (kg/m^2^)			
≥25/<25	1.472	4.356 (1.399–13.563)	0.011

IBL = intraoperative blood loss; BMI = body-mass index; CI = confidence limits

P < .05 was considered statistically significant

**Table 6:** Logistic regression analysis was performed to evaluate predictive factors for high IBL

Variable	Coefficient	Odds ratio (95% CI*)	*p *value
Age	0.054	1.055 (1.004–1.109)	0.035
Operative procedure			
TG/DG	1.404	4.071 (1.626–10.193)	0.003
VFA (cm^2^)			
≧100/<100	1.154	3.170 (1.251–8.033)	0.015

IBL = intraoperative blood loss; VFA = visceral fat area; CI = confidence limits

P < .05 was considered statistically significant

## Discussion

This study revealed that a high BMI adversely affected the IBL, and that age, operative procedure and BMI or VFA independently affected high IBL in open gastrectomy with D2 lymph-node dissection.

Gastric cancer is one of the most common malignancies in Japan [14]. Dietary changes favouring Western-style eating habits have resulted in an increased rate of obesity in the Japanese population [13]. Surgeons have thus had more opportunities to treat obese patients with gastric cancer in recent years.

Surgeons often assume that obese patients will suffer from adverse effects with respect to the short-term surgical outcomes of gastric surgery. Several studies have discussed the influence of obesity on surgical morbidity in gastric surgery. Some reported that high BMI was associated with increased intraoperative and postoperative morbidity in open D2 gastrectomy [1,2,15]. By contrast, others reported that high BMI had no effect on undesirable surgical outcomes of open gastric surgery [16,17].

The current study evaluated the surgical outcomes of open gastrectomy with D2 lymph-node dissection for gastric cancer according to BMI. High BMI had a significant adverse effect on the IBL, but not on the operating time, the number of dissected lymph nodes or the morbidity, owing to careful perioperative management. The volume of IBL was thought to be greater in the high BMI group because fatty tissue has more abundant blood vessels; leakage from the fatty tissue might thus have accounted for the relatively high blood loss. The morbidity, however, remained relatively low within the high BMI group.

The BMI represents both the SFA and the intraperitoneal fat area. A recent report revealed that the VFA/body surface area might be a more useful index than the BMI for predicting the technical difficulties involved in laparoscopic resection of rectosigmoid carcinoma [18]. However, to our knowledge, no previous reports have assessed the influence of VFA on the short-term surgical outcomes of open gastrectomy with D2 lymph-node dissection. The current study showed that patient age, operative procedure and BMI or VFA could independently predict high IBL. These results suggest that the VFA is a useful predictive factor for high IBL in open gastrectomy D2 lymph-node dissection. Moreover, the BMI might be superior to the VFA, because it is simpler to measure.

An excess of fatty tissue necessitates more complex lymph-node dissection and a larger cutting area, which can sometimes be associated with haemorrhaging. More delicate haemostatic manipulation is thus necessary in obese patients to reduce the volume of IBL.

## Conclusion

It will be necessary to perform operative manipulations more carefully in patients with high BMI or high VFA, and to develop new devices with better haemostatic functions, in order to reduce blood loss during D2 gastrectomy.

## List of abbreviations

BMI: body-mass index; CT: computed tomography; IBL: intraoperative blood loss; JGC: Japanese Classification of Gastric Carcinoma; ODG: open distal gastrectomy; OR: odds ratio; OTG: open total gastrectomy; SD: standard deviation; SFA: subcutaneous fat area; VFA: visceral fat area.

## Competing interests

The authors declare that they have no competing interests.

## Authors' contributions

HM and CK designed the study. HM and CK wrote the manuscript. HM, CK, HA, HAO, TK, and RT performed the screening and classification of published articles. YN, SF, and HS helped with final analysis of the data and editing of the manuscript. All authors read and approved the final manuscript.
